# Endocannabinoid Modulation of Nucleus Accumbens Microcircuitry and Terminal Dopamine Release

**DOI:** 10.3389/fnsyn.2021.734975

**Published:** 2021-08-23

**Authors:** Dan P. Covey, Alyssa G. Yocky

**Affiliations:** Department of Neuroscience, Lovelace Biomedical Research Institute, Albuquerque, NM, United States

**Keywords:** dopamine, nucleus accumbens, endocannabinoids, circuitry, optogenetics

## Abstract

The nucleus accumbens (NAc) is located in the ventromedial portion of the striatum and is vital to valence-based predictions and motivated action. The neural architecture of the NAc allows for complex interactions between various cell types that filter incoming and outgoing information. Dopamine (DA) input serves a crucial role in modulating NAc function, but the mechanisms that control terminal DA release and its effect on NAc neurons continues to be elucidated. The endocannabinoid (eCB) system has emerged as an important filter of neural circuitry within the NAc that locally shapes terminal DA release through various cell type- and site-specific actions. Here, we will discuss how eCB signaling modulates terminal DA release by shaping the activity patterns of NAc neurons and their afferent inputs. We then discuss recent technological advancements that are capable of dissecting how distinct cell types, their afferent projections, and local neuromodulators influence valence-based actions.

## Introduction

Accurate valence-based predictions and appropriate action sequences are necessary for survival, and disruptions in this process underlie numerous neuropsychiatric disorders. The nucleus accumbens (NAc) is a brain region that is central to valence-based predictions and goal-directed actions, and neuropsychiatric treatments often target neural signaling in the NAc (e.g., Deep Brain Stimulation (DBS), pharmacotherapies). State-of-the-art neuroscience techniques capable of interrogating cell type- and anatomically specific neural elements are continuously clarifying how NAc function controls stimulus encoding and goal-directed behavior. Here, we will discuss ongoing efforts aimed at elucidating NAc control of behavior and propose critical, outstanding questions.

The NAc is a relatively small region of the ventral striatum that relays input from cortical and subcortical “limbic” brain regions onto basal ganglia motor circuits (Groenewegen et al., 1987; Klawonn and Malenka, 2018), allowing emotion to influence action (Mogenson et al., 1980; Floresco, 2015). Dysfunction in this region is also involved in numerous neuropsychiatric disorders including addiction, depression, and chronic stress (Russo and Nestler, 2013; Rudebeck et al., 2019). Seminal work aimed at understanding the function and dysfunction of the NAc has identified separate cell populations based on molecular profile, electrophysiological properties, or output target that differentially influence valence-based behaviors (Gerfen and Surmeier, 2011; Lobo and Nestler, 2011). This classification system of striatal neurons has massively influenced our understanding of the neurobiology of motivated behavior, disease, and its treatment (Alexander et al., 1986; Albin et al., 1989). However, this overall framework implies separate homogenous cell populations that do not exist in the NAc. Rather than distinct cell populations defining specific circuits that serve separate functions, the NAc controls behavior through coordinated interactions between complex microcircuits, consisting of diverse cell types with different afferent inputs and output targets. For the purposes of this review, we will focus on how neuromodulatory systems interact with local NAc microcircuitry and associated networks to influence valence processing and associated actions.

Another common misconception is that the NAc functions as a “reward center.” Support for this idea is based on evidence that disruptions in NAc function through experimental manipulation or disease diminish goal-directed action and predictably reduce reward seeking (Ungerstedt, 1976; Zhou and Palmiter, 1995). Nevertheless, targeted inactivation of the NAc using lesions or pharmacology does not disrupt hedonic reactions (Berridge, 2007) or appetitive responding when the reinforcement contingency is simple or reward cost is low (Parkinson et al., 2002; Corbit and Balleine, 2011). Moreover, the NAc is required for overcoming physical or cognitive costs in pursuit of reward (Salamone and Correa, 2002) or to avoid aversive stimuli (Pezze and Feldon, 2004; Klawonn and Malenka, 2018). Thus, rather than a “reward-region,” the NAc appears to more generally allow stimuli associated with motivationally salient events to invigorate the initiation and continuation of approach or avoidance behavior. The neural mechanisms that mediate this rather broad and still somewhat poorly defined role remain difficult to pinpoint due to the highly integrative architecture of the NAc, receiving input from cortical and subcortical regions that is filtered by complex interactions within the NAc.

In this review, we will focus on two important filters of NAc function: dopamine (DA) and endocannabinoid (eCB) neuromodulators. DA release in the NAc controls motivated action (Salamone and Correa, 2002) and DA dysfunction in this region contributes to numerous neurological and neuropsychiatric disorder that are characterized by aberrant forms of goal-directed behavior (Russo and Nestler, 2013). Additionally, alterations in NAc eCB signaling are indicated in a variety of disorders (Araque et al., 2017; Patel et al., 2017). As reviewed previously (Oleson et al., 2012; Covey et al., 2014, 2015, 2017), eCB signaling modulates midbrain DA neuron activity to shape downstream DA concentration changes in the NAc during goal-directed action. Here, we will discuss ongoing work investigating how eCB signaling influences neural circuitry within the NAc to locally shape terminal DA release. Specifically, we will cover the complex neural architecture of the NAc (section “Neural Architecture of the NAc”) and how eCB signaling modulates interactions between various NAc cell types (section “Endocannabinoid Control of NAc Microcircuits”) to locally influence DA release (section “DA Signaling in the NAc”). We conclude that an understanding of how the NAc controls action cannot be accomplished by isolating individual components of NAc function (e.g., cell firing, receptor binding), but by dissecting how various cell types, their afferent projections, and local modulators interact to control complex behaviors. We end by discussing recent technological advancements that will facilitate this endeavor (section “Conclusions, Caveats, and Future Directions”).

## Neural Architecture of the NAc

The NAc is bound medially by the septum, ventrally by the olfactory tubercle, and extends rostrally from the bed nucleus of the stria terminalis to the rostral pole (Zahm and Brog, 1992). The NAc is generally subdivided into the more dorsal core (NAcC) and ventral shell (NAcSh) subregions (Zaborszky et al., 1985) that can be immunohistochemically distinguished based on expression of several proteins, including calcium-binding protein calbindin D28k (core > shell), acetylcholinesterase (shell > core), and substance P (shell > core) (Zahm and Brog, 1992). The cellular architecture of the NAc consists primarily of medium spiny neurons (MSNs), which constitute ∼95% of the cell types in the NAc and 100% of the projection neurons (Meredith et al., 1993; Meredith, 1999). Although small in number relative to MSNs, separate classes of NAc interneurons with distinct molecular profiles and electrophysiological properties exert a profound influence on NAc cell excitability and terminal neurotransmitter release. We will provide a brief overview of each neuron population below but refer readers to excellent reviews for a more detailed discussion (Tepper et al., 2018; Castro and Bruchas, 2019; Robinson and Thiele, 2020; Schall et al., 2021).

### Medium Spiny Neurons (MSNs)

Medium spiny neurons–or spiny projection neurons (SPNs)–are named as such based on their medium size (∼15 um diameter) and dendritic arbors that are covered in small membrane protrusions [i.e., spines; (Kawaguchi, 1993; Meredith, 1999)]. MSNs exhibit low baseline firing rates (∼1 Hz) *in vivo* (O’Donnell et al., 1999) and are resistant to excitation due to intrinsic properties combined with afferent control. NAc MSNs possess an inward rectifying K^+^ conductance and hyperpolarized resting membrane potential (−70 to −85 mV) and are subject to substantial tonic inhibition from GABAergic interneurons. Thus, strong excitatory input, is required to reach spike threshold and drive MSN output. This cellular architecture is conceptualized as a filter that allows transmission of only the most salient information onto downstream motor nuclei of the basal ganglia.

Medium spiny neurons are segregated into two populations according to whether they express D1- or D2-type DA receptors (Gerfen and Surmeier, 2011; Lobo and Nestler, 2011). D1- and D2 type receptors are G-protein couple receptors (GPCRs) that differ in their downstream signaling cascades. D1-type receptors (including D1 and D5) are “excitatory” in that they positively couple to Gα_*s*_ and stimulate adenylyl cyclase (AC) signaling cascades. Conversely, D2-type receptors (including D2, D3, and D4) are “inhibitory” in that they positively couple to Gα_*i/o*_ to inhibit AC production and reduce cell excitability (Lachowicz and Sibley, 1997). It should be noted that DA receptor binding has no direct effect on cell firing, but modulates how synaptic transmission alters cell function by positively or negatively influencing voltage-dependent conductance. Both MSN populations are distributed equally throughout most of the NAc, although parts of the medial NAcSh express relatively low-levels of D2-MSNs (Gangarossa et al., 2013). Few striatal MSNs express both receptors, but D1/D2 co-expression increases along a ventromedial gradient from 7.3% in NAcC to ∼14.6% in NAcSh (Surmeier et al., 1996; Gagnon et al., 2017). As we discuss below (see section “Non-canonical Neuromodulator Control of DA Release”), the two MSN populations can also be distinguished based on neuropeptide signaling mechanisms.

In addition to DA receptor expression, MSNs are also differentiated by their projection target. D1-MSNs preferentially project to the ventral mesencephalon, including dense synapses onto GABA and DA neurons in the ventral tegmental area (VTA). In contrast, D2-MSNs preferentially target GABA neurons in the ventral pallidum (VP), which creates an indirect pathway to the VTA (Lobo and Nestler, 2011). These dichotomous pathways are akin to the “direct” and “indirect” pathways in the dorsal striatum that define the basal ganglia motor loops (Gerfen and Surmeier, 2011; Kravitz et al., 2012). Molecular and anatomical differences are often proposed to allow D1- and D2-MSNs in the NAc to exert opposing control over behavior. In support of this model, optogenetic activation of NAc D1-MSNs facilitates a cocaine conditioned place preference (CPP) (Lobo et al., 2010; Koo et al., 2014), whereas chemogenetic inhibition of D1-MSNs (Calipari et al., 2016) or D2-MSN activation suppresses cocaine CPP (Lobo et al., 2010; Koo et al., 2014). Moreover, D2-MSN signaling is required for avoidance learning and D1-MSNs support reward learning (Hikida et al., 2010, 2013).

The functional distinction between D1- and D2-MSN populations–D1 projections to the midbrain promote approach while D2 projections to the VP facilitate avoidance–is supported by numerous studies. However, this approach/avoidance segregation between MSN populations is over-simplified. First, while D1-MSNs projecting from the NAcC to the VTA express only the D1 receptor, ∼50% of D1-MSNs also project to the VP along with D2-MSNs (Lu et al., 1998; Kupchik et al., 2015; Soares-Cunha et al., 2020). Activity between the two populations is also interconnected through local GABAergic collaterals: D1-MSNs project onto other D1-MSNs but D2-MSNs project onto both D1- and D2-MSNs (Taverna et al., 2007). Additionally, D1 and D2-MSN activity–based on fiber photometry measurements of Ca^2+^ signaling–are dramatically different in the medial NAcC during a cocaine CPP task (Calipari et al., 2016), while Ca^2+^ signaling in the lateral NAc during an operant lever pressing task is strikingly similar between D1- and D2-MSNs (Natsubori et al., 2017). Thus, D1- and D2-MSN activity levels exhibits regional and context-dependent similarities and differences, rather than a dichotomous profile/phenotype.

Moreover, optogenetic manipulation studies are inconsistent (Table 1). Notably, both D1- and D2-MSNs (Soares-Cunha et al., 2016) and their projections to the VP (Soares-Cunha et al., 2018), facilitate the motivation to work for sucrose reinforcement (Soares-Cunha et al., 2016). Moreover, both populations reinforce behavior, as measured with optogenetic intracranial self-stimulation (ICSS), although reinforcement rate is much greater in D1 versus D2 MSNs (Cole et al., 2018). Alternatively, optogenetic activation of D1-MSNs supports a CPP, while D2-MSN activation has no effect (Cole et al., 2018) or is aversive (Soares-Cunha et al., 2020). However, elegant work by Soares-Cunha et al. (2020) demonstrates that optogenetic activation of both D1- and D2-MSNs supports a place preference or aversion, depending on stimulation parameters. Overall, rather than exerting diametric control over goal-directed behavior by driving approach or avoidance, both MSN populations support a more nuanced and overlapping role in various aspects of motivated actions that is unlikely to be recapitulated by bulk measures or manipulations of population-level signaling dynamics during rudimentary behavioral assays (e.g., place preference, fear conditioning).

**TABLE 1:** Optogenetic manipulations of NAc neurons.

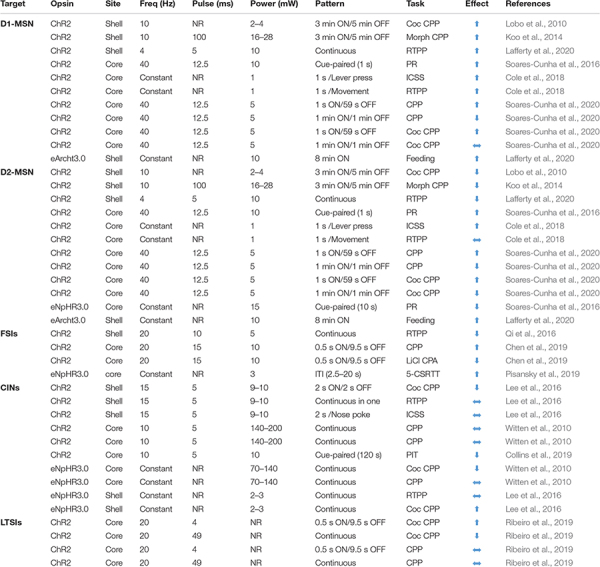

*Shown are the targeted neuron populations (Target), Opsin, NAc subregion (Site), optogenetic experimental parameters (Opsin), Frequency (Freq), Pulse width (Pulse), Light power (Power), and Stimulation (Pattern), behavioral task (Task), and behavioral effect (Effect; Increase: 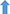, Decrease 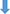:, No Effect: 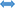). Reported values are from each reference (Ref), unless values were not reported (NR). Behavioral tasks include drug conditioned place preference (CPP), real-time place preference (RTPP), and intracranial self-stimulation (ICSS) to assess hedonic processing or positive reinforcement; progressive ratio (PR) operant schedule to assess motivation; lithium-chloride conditioned place aversion (LiCl CPA) to assess avoidance; 5-choice serial reaction time task (5-CSRTT) to assess impulsivity; and Pavlovian-to-instrumental transfer (PIT) to assess conditioned motivation.*

### Excitatory Inputs

**FIGURE 1:**
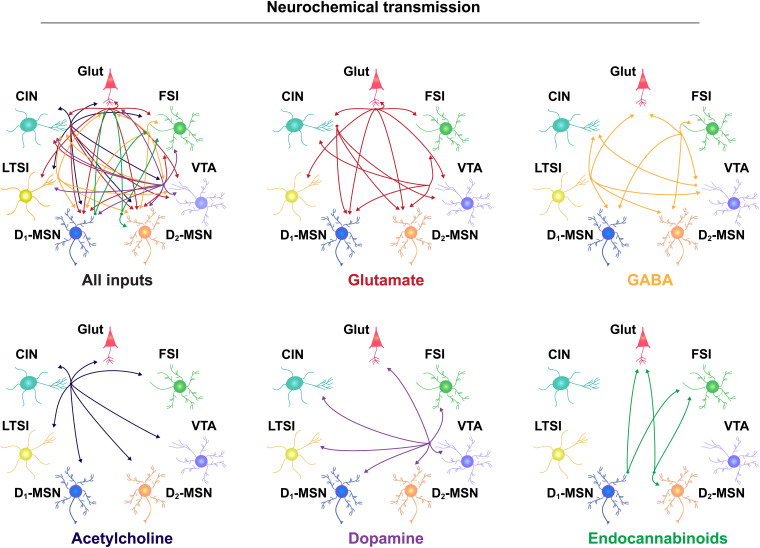
Neurochemical communication in the NAc. Neurotransmitter release from separate cellular populations in the NAc, including glutamatergic (Glut) neurons from throughout the brain, fast-spiking interneurons (FSIs), the ventral tegmental area (VTA), D2- and D1-medium spiny neurons (MSNs), low-threshold spiking interneurons (LTSIs), and cholinergic interneurons (CINs). Input-output relationship is depicted by the direction of the arrow and separated according to neurotransmitter molecule, listed below each diagram.

**FIGURE 2:**
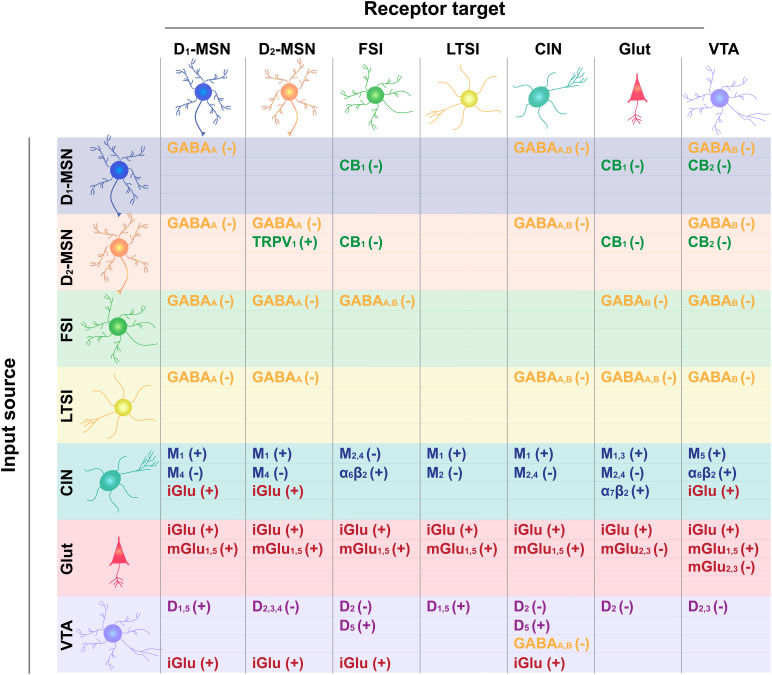
Receptor target for neurotransmitter input-output connections depicted in Figure 1. Input cell type is shown on the left and output target on the top. GABA type A and B receptor targets are shown in yellow, cannabinoid type 1 (CB1) and 2 (CB2) receptor targets are in green, glutamate ionotropic (iGluR) and metabotropic (type 2,3: mGluR_2,3_; type 1,5 mGluR_1,5_) are in red, acetylcholine muscarinic receptor subtypes (M_1_; M_2_; M_4_; M_1,3_; M_2,4_; M_5_) and ionotropic receptor subtypes (alpha 6 Beta 2, α_6_β_2_ and α_2_β_2_) are in blue, and DA receptor subtypes (D_1_-type: D_1_, D_5_, D_1/5_; D_2_-type: D_2_, D_2/3_, D_2/3/4_) are in purple. How receptor binding influences cell excitability is indicated by a positive (+) or negative (–) sign.

As noted above, action potential generation in NAc MSNs relies on excitatory input. Glutamatergic projections arise from numerous regions including, but not limited to, anterior cortical areas, amygdala, hippocampus, and thalamus (Goto and Grace, 2005; Sesack and Grace, 2010; Russo and Nestler, 2013). For the purposes of this review, we will focus our discussion on the more widely studied excitatory NAc projections arising from the prefrontal cortex (PFC), basolateral amygdala (BLA), and ventral hippocampus (vHPC). Anatomically distinct glutamatergic inputs to the NAc are proposed to relay distinct forms of environmental information; the vHPC encodes contextual information, the BLA relays emotionally salient events, and the PFC signals value (Everitt and Wolf, 2002; Kelley, 2004; Pennartz et al., 2011; Russo and Nestler, 2013). Inputs are also anatomically distributed across the NAc; cortical areas preferentially innervate the NAcC, while the vHPC preferentially projects to the NAcSh, and the BLA projects equally to both (Britt et al., 2012; Li Z. et al., 2018; Deroche et al., 2020). However, there is striking overlap in the density and behavioral influence of these glutamatergic inputs. First, all regions similarly innervate D1- and D2-MSNs (Barrientos et al., 2018; Li Z. et al., 2018). Moreover, optogenetic activation of NAc inputs from either PFC, vHPC, or BLA is reinforcing, as measured by ICSS (Britt et al., 2012; Mateo et al., 2017). The activity patterns of these separate NAc inputs (as measured by fluorescent Ca^2+^ sensors) are also strikingly similar during reward-seeking tasks, and their optogenetic activation similarly reduces food consumption (Reed et al., 2018). Accordingly, and for the sake of simplicity, we depict each region as a single glutamate source in our NAc functional connectivity map (Figure 1) and receptor interaction diagram (Figure 2).

Despite the general functional overlap among glutamatergic inputs to the NAc, heterogeneity emerges in the input-output connections of separate projections. Deroche et al. (2020) found that excitatory synaptic strength–as measured by the amplitude of optogenetically evoked excitatory post-synaptic currents (oEPSCs) or the probability of post-synaptic action potential generation–varied according to the afferent input and target subpopulation. Excitatory synaptic strength from the BLA was greatest onto D1-MSNs, while the PFC and vHPC exhibited stronger inputs to D2-MSNs (Deroche et al., 2020). However, separate work using similar approaches found substantially stronger inputs from the vHPC onto D1-MSNs (MacAskill et al., 2012; Scudder et al., 2018) due to more proximal synaptic connections at D1- versus D2-MSN dendrites (MacAskill et al., 2012). Because optogenetic excitation often relies on viral transduction, contrasting findings may arise due to slight differences in injection site or viral infectivity. Moreover, genetically- and functionally-distinct subpopulations of glutamatergic inputs arising from the same projection region differentially affect behavior. Optogenetic activation of BLA excitatory projections to the NAc following expression of ChR2 under the regulatory elements of the CaMKIIα promoter is reinforcing (Stuber et al., 2011). However, Shen et al. (2019) recently identified a separate, non-overlapping population of BLA to NAc glutamatergic projections that express the neuropeptide cholecystokinin (CCK) (Shen et al., 2019). These CCK-expressing projections from the BLA preferentially innervate D2-MSNs in the NAc and optogenetic activation of this circuit is aversive, highlighting the critical importance of dissecting the anatomical and molecular properties of separate NAc circuits. As will be discussed below (section “Endocannabinoid Control of NAc Microcircuits”), the strength of input- and output-specific NAc projections are also distinguished based on their regulation by eCB signaling.

### GABAergic Interneurons

One GABAergic interneuron population is often distinguished based on the expression of the calcium binding protein parvalbumin (PV) and are therefore referred to as PV interneurons. We will use the term fast-spiking interneurons (FSIs) here because a population of GABAergic NAc interneurons display electrophysiological properties characteristic of FSIs (see section “Endocannabinoid Control of NAc Microcircuits”) and release GABA, but do not express PV (Winters et al., 2012; Schall et al., 2021). Similar to MSNs, NAc FSIs are “medium” sized (Kawaguchi, 1993), lack spontaneous activity *in vitro*, and rely on excitatory input from similar brain regions for action potential generation, including the PFC (Yu et al., 2017), BLA (Yu et al., 2017), vHPC (Yu et al., 2017; Scudder et al., 2018; Trouche et al., 2019), and VTA (Qi et al., 2016). However, excitatory synaptic strength is much greater onto FSIs compared with neighboring MSNs (Wright et al., 2017; Yu et al., 2017; Scudder et al., 2018), resulting in high maximal firing rates, upward of ∼150 Hz (Taverna et al., 2007), and sustained feedforward inhibition of target MSNs *in vivo*. While lateral inhibition from MSN collaterals is prominent in the dorsal striatum (Chuhma et al., 2011), FSIs provide the major source of inhibitory control in the NAc (Qi et al., 2016; Wright et al., 2017; Yu et al., 2017; Schall et al., 2021). FSIs also form electrical and chemical FSI-to-FSI connections (Winters et al., 2012), allowing synchronized and widespread inhibitory control over both D1- and D2-MSNs (Scudder et al., 2018). Notably, FSIs do not make synaptic contacts with low threshold spiking interneurons (LTSIs) or cholinergic interneurons (CINs) (Straub et al., 2016). Recent work shows that FSIs also gate NAc function via GABA_*B*_ receptors located on glutamatergic inputs that preferentially synapse onto D1-MSNs (Manz et al., 2019). Whether this GABA_*B*_-mediated inhibition differs across glutamatergic projections or post-synaptic interneuron populations is not clear. As will be discussed below (section “Endocannabinoid Control of NAc Microcircuits”), FSIs can be further distinguished as the only NAc neuronal population that expresses the cannabinoid type 1 (CB1) receptor within the NAc (Winters et al., 2012).

While FSIs provide robust inhibitory control over NAc neuronal activity, establishing how FSIs influence behavior has been difficult. Qi et al. (2016) found that direct optogenetic activation of NAc PV-expressing FSIs or their glutamate inputs, promotes place aversion. In contrast, Chen et al. (2019) found optogenetic activation of this neuronal population elicits a place preference and their inhibition is aversive. Discrepancies between these two studies may be ascribed to different optogenetic stimulation protocols (Table 1). Altogether, differences across studies highlight that behaviorally relevant patterns of FSI activity–which are highly dynamic and uncoordinated during reward-seeking tasks (Berke, 2008)–may be difficult to recapitulate using synchronous, population-wide activation or inhibition.

A second GABAergic interneuron population are the LTSIs. Similar to FSIs, LTSIs are medium-sized (9–24 um) neurons that lack dendritic spines (i.e., “aspiny”), receive afferent input from similar regions as MSNs (Ribeiro et al., 2019), and control information flow into and out of the NAc via local GABAergic modulation. LTSIs are named as such based on their low-threshold Ca^2+^ spike that, in conjunction with their relatively depolarized membrane potential (∼−50 mV) and spontaneous activity patterns, supports a highly excitable neuronal population (Scudder et al., 2018; Tepper et al., 2018; Robinson and Thiele, 2020). Striatal LTSIs send long axonal projections (up to 1 mm) that synapse onto distal dendrites of D1- and D2-MSNs and CINs (Straub et al., 2016). Similar to FSIs, LTSIs do not show biased input onto D1- versus D2-MSNs (Scudder et al., 2018). This anatomical arrangement allows LTSIs to influence NAc output over large distances, but their effect on MSN spiking activity is weaker compared to the more proximal synapses formed by FSIs. In addition to GABA, LTSIs are also highly enriched in the neuropeptides somatostatin (SOM), the neuropeptide Y (NPY) receptor, and nitric oxide synthase (NOS), and are often categorized as the SOM/NPY/NOS+ interneurons (Kawaguchi, 1993; Tepper et al., 2018).

Targeted manipulations of LTSI subpopulations indicate an important role in motivated behavior. Optogenetic activation of SOM-expressing NAc neurons enhances a cocaine CPP, which is suppressed by optogenetic inhibition (Ribeiro et al., 2019). Chemogenetic stimulation of NOS-expressing interneurons in the NAc facilitates the acquisition rate of cocaine self-administration (Smith et al., 2017) and sucrose reinforcement (Bobadilla et al., 2017). Finally, NPY infusions into the NAc produce a CPP (Brown et al., 2000), elevate extracellular dopamine levels measured with microdialysis (Sorensen et al., 2009), and increase motivation to seek and consume sucrose (van den Heuvel et al., 2015). As noted above, SOM, NOS, and NPY are typically expressed in the same LTSI cells along with GABA (Tepper et al., 2018). Thus, manipulations that use any one of these molecular markers to target one subpopulation of LTSIs will likely alter multiple modes of neuromodulatory signaling. Moreover, common optogenetic approaches are optimized for manipulating target populations on fast timescales that may not mimic endogenous neuropeptide or gaseous transmitter signaling.

### Cholinergic Interneurons (CINs)

Cholinergic interneurons can be distinguished from other NAc neuron populations by their much larger cell bodies (∼20–30 um diameter), long (up to 1 mm) axonal branches, and expression of the acetylcholine (ACh) synthesizing enzyme choline acetyltransferase. Striatal CINs rest at a relatively depolarized potential (∼−60 mV) (Bennett et al., 2000; Gonzales and Smith, 2015) and exhibit tonic firing patterns (∼2–10 Hz) (Zhou et al., 2002). Accordingly, CINs are also referred to as “Tonically Active Neurons” (TANs). Afferent input to CINs arises from similar extra-striatal regions as MSNs and local LTSIs, but not from FSIs or other CINs (Guo et al., 2015). Notably, the majority of synaptic connections onto striatal NAc CINs are GABAergic (Gonzales and Smith, 2015), including extrinsic GABAergic input from the VTA (Brown et al., 2012).

Cholinergic interneurons exert complex actions on NAc microcircuitry through ACh release onto various cholinergic receptor subtypes, including ionotropic nicotinic receptors (nAChRs) and metabotropic muscarinic receptors (mAChRs) that are located on axon terminals and somatodendritic compartments (Gonzales and Smith, 2015). nAChRs form pentameric ion (Na^+^, K^+^, and Ca^2+^) channels consisting of a combination of α (α2–α10) and β (β2–β4) subunits, with the α6/β2 variety forming the predominant type in the NAc (Zhou et al., 2002; Exley and Cragg, 2008). Analogous to D1- and D2-MSNs, mAChRs can be separated into two categories; G_*q/11*_-coupled M1-like receptors (M1, M3, and M5) that enhance internal calcium release through stimulation of phospholipases, and the G_*i/o*_-coupled M2-like receptors (M2 and M4) that block calcium channel activity by reducing cyclic AMP formation through the inhibition of adenylyl cyclase (Gonzales and Smith, 2015). Moreover, the vast majority of CINs express the vesicular glutamate transporter-3 (vGlut3) and can release glutamate onto both ionotropic and metabotropic glutamate receptors in the NAc (Nelson et al., 2014; Mateo et al., 2017).

The variety of receptor subtypes and binding sites allows CINs to exert direct or indirect excitatory and inhibitory effects on NAc circuitry via pre- or post-synaptic targets. For example, CINs can excite output targets via nAChRs on glutamatergic, GABAergic, or dopaminergic neurons (Nelson et al., 2014; Mateo et al., 2017) or through M1 mAChRs expressed on MSNs and GABAergic interneurons (Abudukeyoumu et al., 2019). Accordingly, feedforward excitation or inhibition is possible through potentiation of glutamate or GABA signaling, respectively. Witten et al. (2010) found optogenetic excitation of NAc CINs inhibited MSN firing via nAChR-dependent activation of GABA release, although the source of GABAergic input is unclear. Alternatively, Mateo et al. (2017) found that CIN activation elicited a nAChR-dependent activation of PFC glutamatergic input to MSNs. In contrast, binding the inhibitory M2/M4 receptors on axon terminals can suppress pre-synaptic neurotransmitter release from glutamatergic, GABAergic, or dopaminergic terminals, and from CINs themselves (Threlfell et al., 2012; Shin et al., 2017), leading to an inhibitory or dis-inhibitory effect. Moreover, by acting as a diffuse, volume transmitter that coordinates activity over large distances, CIN-evoked acetylcholine release can simultaneously affect numerous receptor systems in the NAc (Gonzales and Smith, 2015).

Neural manipulation of NAc CINs support a crucial role in reward-related behaviors, although the direction of this effect is unclear (Table 1). Optogenetic activation of NAc CINs elicits DA release (Mateo et al., 2017), which generally promotes reinforcement. Yet, optogenetic excitation or inhibition of NAc CINs does not support a place preference, *per se*, but can alter a cocaine CPP. Optogenetic inhibition both suppresses (Witten et al., 2010) and facilitates (Lee et al., 2016) a cocaine CPP, while optogenetic excitation augments a cocaine CPP (Lee et al., 2016) or has no effect (Witten et al., 2010). Suppression of CIN activity using a membrane-tethered toxin against voltage-gated calcium channels leads to anhedonic behaviors, defined as a decrease in sucrose preference and immobility (Warner-Schmidt et al., 2012) and chemogenetic inhibition of NAc CINs suppresses the ability of rewards (food, social interaction, and cocaine) to increase NAc DA release measured by microdialysis (Hanada et al., 2018). As we discuss further in section “Endocannabinoid Control of NAc Microcircuits,” the relationship between CIN activity, NAc DA release, and valence-driven behavior remains unclear, but is an intense area of investigation.

## Endocannabinoid Control of NAc Microcircuits

The eCB system–consisting of fatty acid signaling molecules, their synthetic and degradative enzymes, and constituent receptors–is a vast signaling network that controls synaptic transmission throughout the brain and periphery (Katona and Freund, 2012; Covey et al., 2017). The primary site of eCB action in the brain is the CB1 receptor, which functions to suppresses pre-synaptic neurotransmitter release via inhibition of Ca^2+^ influx through voltage-gated Ca^2+^ channels (VGCCs), inhibition of adenylyl cyclase (AC), and activation of inwardly rectifying K^+^ channels. CB1 receptor expression exhibits a decreasing dorsolateral to ventromedial gradient in the striatum, with relatively sparse expression in the NAc (Herkenham, 1992; Pickel et al., 2006). Nevertheless, NAc CB1 receptors are critical to short- and long-term forms of synaptic plasticity (Robbe et al., 2002; Grueter et al., 2010) and tightly regulate NAc neurotransmission (Caille et al., 2007) and appetitive behavior (Covey et al., 2017; Mateo et al., 2017). CB1 receptors bind the endogenous fatty acid signaling molecules 2-arachidonoylglycerol (2-AG) and arachidonoyl ethanolamide (AEA, also known as anandamide) (Katona and Freund, 2012; Covey et al., 2017). Both 2-AG and AEA signaling requires their enzymatic synthesis, which is initiated by membrane depolarization, activation of metabotropic receptors coupled to PLCβ (e.g., G_*q/11*_-coupled group 1 metabotropic glutamate receptor-mGluR1/5, muscarinic acetylcholine-mACh-types M1/M3), and increased intracellular [Ca^2+^]. However, important differences in 2-AG and AEA signaling allow dissociable functions.

2-Arachidonoylglycerol is more abundant than AEA in most brain regions, is a full agonist for the CB1 receptor, and is the primary eCB involved in CB1-mediated inhibition of pre-synaptic neurotransmitter release (Ohno-Shosaku and Kano, 2014). This canonical mode of CB1 receptor signaling depends on the *de novo* synthesis and retrograde mobilization of 2-AG from post-synaptic sites onto pre-synaptic CB1-expressing terminals. 2-AG mobilization requires the biosynthetic enzyme, *sn*-1-diacylglycerol lipase-alpha (DGLα), which is expressed in the plasma membrane at dendritic spines of NAc MSNs post-synaptic to CB1 receptor-expressing terminals (Matyas et al., 2007). Cessation of 2-AG signaling occurs primarily through pre-synaptic enzymatic degradation via monoacylglycerol lipase (MAGL), which is localized to the pre-synaptic membrane (Long et al., 2009). Thus, 2-AG levels are determined by the balance between post-synaptic production by DAGLα and pre-synaptic degradation by MAGL.

Arachidonoyl ethanolamide synthesis and degradation mechanisms are less understood. While post-synaptic depolarization and intracellular Ca^2+^ influx support AEA synthesis, the precise mechanisms are unclear (Di Marzo and De Petrocellis, 2012). AEA is synthesized by N-acyl-phosphatidylethanolamine-hydrolyzing phospholipase-D (NAPE-PLD), but alternative synthetic pathways exist (Okamoto et al., 2007). AEA signaling is terminated at post-synaptic sites by the membrane-bound fatty acid amide hydrolase (FAAH) (Cravatt et al., 1996) and potentially through membrane transport via a lipophilic carrier protein (Ronesi et al., 2004). Moreover, AEA is a partial agonist at both the CB1 receptor and Transient Receptor Potential Vanilloid type 1 (TRPV1) channels, non-selective cation channels expressed by D2 MSNs in the NAc (Deroche et al., 2020). Differences in binding affinity may allow AEA to engage CB1 receptors versus TRPV1 channels in a concentration-dependent manner, such that low AEA levels preferentially bind CB1 receptors while higher concentrations affect TRPV1 channels (Di Marzo and De Petrocellis, 2012).

In the NAc, CB1 receptors are expressed on pre-synaptic glutamatergic terminals from the PFC, BLA, and vHPC (Robbe et al., 2002; Grueter et al., 2010; Winters et al., 2012; Wright et al., 2017; Deroche et al., 2020), but not on thalamic inputs (Wu et al., 2015). CB1 receptors are also on inhibitory GABAergic terminals of FSIs (Winters et al., 2012; Wright et al., 2017), but not LTSIs, MSNs, or CINs (Winters et al., 2012; Mateo et al., 2017). This contrasts with the dorsal striatum where CB1 receptors are expressed by both FSI and MSN collaterals (Freiman et al., 2006), and at lower levels on CINs and LTSIs (Fusco et al., 2004). Moreover, while CB1 receptors in the NAc are exclusively expressed by GABAergic neurons with FSI-like electrophysiological properties, ∼50% of these neurons do not express the characteristic PV marker (Winters et al., 2012), which could arguably define another interneuron subpopulation. Collectively, CB1 receptors act within the NAc to suppress excitatory input from throughout the brain and inhibitory input from FSIs.

Cannabinoid type 1-mediated inhibition of pre-synaptic input occurs across a range of time scales. A short-term depression (STD) (lasting tens of seconds) of glutamate input onto MSNs or FSIs and GABAergic input from FSIs onto MSNs occurs following brief (∼5–10 s) post-synaptic depolarization (Winters et al., 2012; Wright et al., 2017; Yu et al., 2017). This eCB-mediated STD (eCB-STD)–termed depolarization-induced suppression of inhibition (DSI) or excitation (DSE)–arises from retrograde eCB mobilization onto pre-synaptic CB1 receptors and allows fine-tuned regulation of ongoing pre-synaptic input. CB1 receptor signaling in the NAc also supports long-term depression (LTD) of CB1-expressing glutamatergic terminals from the PFC, BLA, and vHPC (Robbe et al., 2002; Grueter et al., 2010; Deroche et al., 2020) and GABAergic terminals from CB1-exprressing FSIs (Wright et al., 2017). eCB-LTD at both excitatory and inhibitory synapses depends on post-synaptic Ca^2+^ signaling, activation of the Gq-coupled mGluR5, post-synaptic TRPV1 channel activation, and pre-synaptic CB1 receptor signaling. The functional relevance of receptor-mediated eCB-LTD in the NAc is indicated by studies demonstrating that mGluR5-dependent eCB-LTD is eliminated following exposure to drugs of abuse, presumably due to occlusion (Mato et al., 2005; McCutcheon et al., 2011). Moreover, blocking mGluR5 signaling in the NAc suppresses drug-seeking in a CB1 receptor-dependent manner (Li X. et al., 2018). Accordingly, mGluR5 receptors may represent a promising target for treating substance-abuse and addiction.

Recent work demonstrates that both forms of eCB-mediated plasticity exhibit dramatic heterogeneity according to the projection site and post-synaptic target. Projection-specific ChR2 expression in the PFC, BLA, and vHPC demonstrated greater eCB-STD at BLA inputs on D1-MSNs and PFC to D2-MSNs inputs, while vHPC inputs on D1- and D2-MSNs were equivalent (Deroche et al., 2020). TRPV1 agonism similarly led to inhibition of PFC-evoked oEPSCs in D1- and D2-MSNs, vHPC inputs to D1- but not D2-MSNs, and BLA to D2-MSN inputs while augmenting BLA to D1-MSN inputs (Deroche et al., 2020). Further complexity arises from differential CB1 receptor expression on subpopulations of excitatory inputs arising from the same brain region. The CCK-expressing BLA to NAc projections identified by Shen et al. (2019) (see section “Excitatory Inputs”) also express CB1 receptors (∼90% overlap). These CCK/CB1-expressing projections preferentially synapse onto D2- but not D1-MSNs, and CB1-mediated inhibition of synaptic activity in this pathway supports resilience to the effects of chronic social defeat stress (Shen et al., 2019), a function long-attributed to the NAc (Fox and Lobo, 2019). Continued technological development will be instrumental in further dissecting the immense synapse-specific heterogeneity in the NAc (section “Conclusions, Caveats, and Future Directions”).

## DA Signaling in the NAc

Dopamine neurotransmission in the NAc occurs at *en passant* release sites from dopaminergic axons projecting primarily from the VTA. DA receptors are located on somatic dendrites or axon terminals of all neuron classes in the NAc. The timing and concentration of DA released at target sites arises from a dynamic balance between vesicular release, DA transporter (DAT)-mediated reuptake, and diffusion (Rice and Cragg, 2008). Both release and reuptake are controlled by interactions between membrane excitability, ion channels, G-protein-coupled receptors, and downstream effector molecules, all of which are tightly regulated by local circuit interactions at DA axon terminals (Sulzer et al., 2016; Covey et al., 2017). Recent work argues that NAc DA signaling exerts dissociable effects on behavior depending on the source of modulation, such that cell body-independent mechanisms determine dopaminergic control of motivation, while cell body spiking support DA’s canonical role in reward learning (Mohebi et al., 2019). While there remains little evidence to support such an anatomically dissociable role of DA in controlling behavior, the relationship between terminal DA release and cell body firing has long been recognized as non-linear (Rice and Cragg, 2008), and terminal modulation is a primary factor that dictates how DA neurons ultimately influence NAc function. Yet, how DA terminal modulation controls DA concentration dynamics that associate with or drive discrete behavioral events is poorly understood. Below, we will discuss how prominent afferent projections to the NAc and local microcircuitry interact with eCB signaling to modulate DA release.

### DA Neuron Autoregulation

Dopamine neurons control their own activity through feedback mechanisms at axon terminals via D2 DA autoreceptor signaling and DAT-mediated reuptake. As discussed above (section “Medium Spiny Neurons (MSNs)”), D2 receptors are coupled to inhibitory G_*i/o*_-coupled signaling, thus DA autoreceptors function similarly to pre-synaptic CB1 receptors and suppress pre-synaptic neurotransmitter release, in addition to inhibiting DA synthesis and suppressing vesicular packaging (Ford, 2014). D2 autoreceptors also control DAT function through protein-protein interactions that can increase DAT membrane expression and the rate of DAT-mediated DA reuptake (Lee et al., 2007). Below, we will discuss how these autoregulatory functions interact with a variety of incoming signals that act on receptors expressed by DA terminals to alter DA release via modulation of membrane excitability, DAT function, and DA synthesis.

### Glutamatergic Control of NAc Dopamine Release

As discussed above (section “Excitatory Inputs”), the NAc receives extensive glutamatergic input from throughout the brain. Glutamate acts on both ionotropic (AMPA, NMDA, and kainate) and metabotropic receptors (mGluRs) that are expressed at somatodendritic and axonal compartments throughout the NAc. Eight types of mGluRs are classified into three groups (Niswender and Conn, 2010) that are either G_*q/11*_-coupled (Group 1: mGluR1/5) and stimulate AC and phospholipase C (PLC) signaling cascades or Group 2 (mGluR2/3) and Group 3 (mGluR4/6/7/8) that inhibit AC signaling, Ca^2+^ channels, and activate K^+^ channels through Gα_*i/o*_ signaling. Both mGluR1/5 and mGluR2/3 are densely expressed at both pre- and post-synaptic sites throughout the NAc (Manzoni et al., 1997). Glutamate signaling alters striatal DA release through direct and indirect actions involving ionotropic and mGluRs. Thus, precisely how glutamate input to the NAc alters terminal DA release is unclear.

For example, local application of ionotropic glutamate receptor agonists (kainate, AMPA, and NMDA) inhibits DA release in the NAc (Yavas and Young, 2017) while AMPA receptor antagonists increase DA released in striatal slices, specifically following high intensity stimulation (10 Hz, 3 s) (Avshalumov et al., 2008). This suppression of DA release arises from AMPA receptor-dependent H_2_O_2_ generation in striatal MSNs, which acts as a retrograde messenger to inhibit DA release through activation of K^+^ channels on DA axons (see section “eCB Control of NAc DA Release” below). Synaptic overflow of glutamate during high intensity stimulation can also inhibit DA release via activation of group 1 (Zhang and Sulzer, 2004) or group 2/3 mGluRs (Yavas and Young, 2017) on DA terminals. An inhibitory effect of glutamate receptor activation could also arise through feedforward inhibition via excitation of GABA neurons (see section “GABAergic Control of NAc Dopamine Release”).

In contrast to the inhibitory effect of glutamate, Mateo et al. (2017) recently found that, unlike the dorsal striatum (Chen et al., 1998), DA axons in the NAc express AMPARs and their activation, via local pressure application of AMPA or optogenetic activation of PFC glutamatergic inputs, elicits DA release. However, PFC-evoked DA release remains partly sensitive to nAChR inhibition (Mateo et al., 2017), indicating a contribution from AMPAR-mediated feedforward excitation of NAc CINs (see section “Cholinergic Control of NAc DA Release”). An additional source of glutamatergic modulation arises from VTA projections that co-release DA and glutamate or are exclusively glutamatergic (Zhang et al., 2015). Recent work demonstrates that, similar to other glutamate inputs, VTA glutamatergic projections reinforce behavior independently of DA co-release (Zell et al., 2020).

### GABAergic Control of NAc Dopamine Release

Local regulation of NAc DA release by GABAergic signaling can arise via metabotropic GABA_*B*_ receptors located on DA terminals. However, GABA_*B*_ antagonists do not alter evoked (single- or pulse train) DA release in NAc slices suggesting a lack of tonic GABA_*B*_-mediated inhibition of terminal DA release, although GABAergic tone may be artificially suppressed in slice preparations (Pitman et al., 2014). GABA release from FSIs onto GABA_*B*_ receptor-expressing glutamatergic terminals (Manz et al., 2019) also inhibit DA release. Because ionotropic GABA_*A*_ receptors are not expressed by DA axons (Sulzer et al., 2016), elevated dialysis DA levels following local GABA_*A*_ receptor antagonism (Adermark et al., 2011), or inhibition of DA release in striatal slices by a GABA_*A*_ agonist (Brodnik et al., 2019) arise through an indirect mechanism. Indeed, GABA_*A*_-mediated inhibition of DA release in NAc slices is blocked by a GABA_*B*_ antagonist (Brodnik et al., 2019), indicating that GABA_*A*_ receptors can act upstream to modulate GABAergic output onto GABA_*B*_ receptors on DA axons. This putative circuit remains to be elucidated. Notably, while GABAergic projections from the VTA preferentially synapse onto NAc CINs (Brown et al., 2012), GABA-mediated inhibition of DA release does not rely on striatal CINs (Pitman et al., 2014). Future work is required for elucidating the circuit- and receptor-specific mechanisms by which GABA transmission modulates NAc DA release.

### Cholinergic Control of NAc DA Release

Cholinergic interneurons exert powerful control over dopaminergic transmission in the NAc through extensive axonal branches (section “Cholinergic Interneurons (CINs)”) that are closely intermingled with DA varicosities. ACh release from CINs binds ionotropic nAChRs on DA terminals, which increases intracellular Ca^2+^ flux (Zhou et al., 2002; Exley and Cragg, 2008). Accordingly, NAc CINs are capable of directly eliciting DA release *in vivo*, independently of midbrain neuronal activity (Mateo et al., 2017). Ca^2+^ entry through nAChRs may further potentiate DA release through mobilization of readily releasable pools of vesicular DA (Turner, 2004). However, numerous *in vitro* studies indicate that nAChRs control DA release in a complex manner according to ongoing patterns of DA release, increasing DA release evoked by low-frequency stimulation of DA neurons, and inhibiting DA release during high frequency stimulation (Zhou et al., 2002; Zhang and Sulzer, 2004). Thus, when nAChRs are antagonized or become desensitized by nicotine, DA release evoked by high frequency burst firing patterns is potentiated. In support of this *in vitro* work, NAc infusions of a nAChR antagonist increase cue-evoked DA release and invigorate reward-seeking (Collins et al., 2016), while optogenetic stimulation of NAc CINs during cue presentation suppresses cue-evoked reward seeking. Collectively, the *in vivo* measures are largely in agreement with *in vitro* work; nAChRs on DA terminals facilitate DA release when the DA neuronal firing rate is low, such as in anesthetized animals (Mateo et al., 2017), but suppress DA release during periods of increased DA neuron activity, as occurs during specific epochs of reward-seeking sequences (Collins et al., 2016, 2019). An improved understanding of the relationship between ACh transmission and terminal DA release will likely be afforded by recent advancements in optical imaging approaches that permit rapid ACh and DA detection during behavior (Sabatini and Tian, 2020).

Cholinergic interneurons also target mAChRs, which are of the G_*q/11*_-coupled M5 subtype on DA terminals. Activation of M5 mAChRs in dorsal striatum (Foster et al., 2014) or NAc (Shin et al., 2017) brain slices inhibits DA released by electrical stimulation (Foster et al., 2014), but potentiates DA released by optogenetic activation of DA neurons (Shin et al., 2017). Thus, M5 mAChRs increase DA release through direct actions on DA terminals but can inhibit DA release via a polysynaptic route that is revealed by electrical stimulation. ACh release onto pre-synaptic G_*i/o*_-coupled M4 mACh autoreceptors in the NAc provides another source of regulation by decreasing CIN output onto nAChRs and potentiating DA release evoked by high frequency firing patterns (Threlfell et al., 2012; Shin et al., 2017). mAChR agonists may also potentiate DA overflow by slowing DAT-mediated DA uptake through an unidentified mechanism (Shin et al., 2017). DA release is also modulated by ACh interactions with glutamate signaling, such that NAc DA release is facilitated by ACh acting at nAChRs on glutamate terminals arising from the PFC (Mateo et al., 2017) or VTA (Shin et al., 2017). Finally, a subpopulation of CINs also express the vesicular glutamate tranporter-3 (vGlut-3) and are thus capable of increasing DA release through glutamate co-transmission (Mateo et al., 2017).

### eCB Control of NAc DA Release

Despite the important role of eCB signaling in controlling NAc microcircuitry (section “Endocannabinoid Control of NAc Microcircuits”) and modulating DA input to the NAc (Oleson et al., 2012; Covey et al., 2015, 2017), our understanding of how eCBs influence terminal DA release is limited. Because DA neurons do not express CB1 receptors (Julian et al., 2003), the ability of CB1 receptor manipulations to control NAc DA release and motivated action (Oleson et al., 2012; Wang et al., 2015; Covey et al., 2018) has generally been ascribed to CB1-mediated changes in pre-synaptic input onto midbrain DA neurons projecting to the NAc. However, mounting evidence demonstrates that CB1 receptors also control DA release at the level of NAc terminals. For example, voltammetry recordings in striatal brain slices found that CB1 agonists inhibit DA release following pulse-train stimulation (10 Hz, 3 s) of striatal DA terminals (Sidlo et al., 2008). Thus, CB1 receptors can exert a similar effect as nAChRs and H_2_O_2_ to suppress DA release evoked by more intense stimulations. The CB1-mediated inhibition of DA release depends on H_2_O_2_-mediated activation of K_*ATP*_ channels in DA terminals (Sidlo et al., 2008). However, the precise mechanisms by which CB1 receptors control DA release, including the site of eCB production and receptor binding, remains unclear.

Recent work identified a complex circuit by which eCBs locally control NAc DA release (Mateo et al., 2017). In this study, optogenetic stimulation of CINs elicited NAc DA release recorded with FSCV in anesthetized mice and in brain slices. Moreover, a CB1 agonist was found to inhibit CIN-evoked DA release, although the site of CB1 receptor action is not readily apparently because, as mentioned above, neither CINs nor DA neurons express CB1 receptors. Rather, it was found that CIN stimulation facilitates glutamatergic transmission via pre-synaptic α7-expressing nAChRs located on PFC terminals. Increased glutamate release, in turn, drives DA release through at least two mechanisms: (1) directly via glutamate release onto AMPA receptors located on DA terminals or (2) indirectly through excitation of CINs and activation of nAChRs on DA terminals. In the NAc, CIN-evoked eCB production also occurs through facilitation of glutamate release onto NAc MSNs, which drives eCB mobilization onto CB1 receptor-expressing PFC terminals. Behavioral relevance for CB1 regulation of this pathway was obtained by assessing ICSS for optogenetic stimulation of PFC to NAc inputs, which has previously been shown to support high rates of reinforcement (Britt et al., 2012). ICSS rates were suppressed by inhibiting degradation of the endogenous CB1 receptor ligand 2-AG with an MAGL inhibitor. Notably, an opposite effect on reinforcement rates is observed following MAGL inhibition when behavior is maintained by stimulation of the midbrain (Oleson et al., 2012) or sucrose reinforcement (Covey et al., 2018), highlighting site-specific control of reinforcement by eCB signaling. Moreover, ICSS rates were augmented by pathway-specific CB1 receptor deletion, presumably due to loss of inhibitory feedback onto PFC terminals. Collectively, this work demonstrates that NAc CIN activation evokes DA release, in part, through excitation of CB1 receptor-expressing PFC glutamate terminals.

While the work by Mateo et al. (2017) elegantly dissects complex mechanisms by which eCBs and NAc microcircuitry interact to shape NAc DA release, the behavioral relevance of this circuit remains to be fully elucidated. NAc nAChR signaling suppresses DA release during periods of heightened DA neuron activity (section “Cholinergic Control of NAc DA Release”) and in response to motivationally salient stimuli during reward seeking (Collins et al., 2016, 2019). Moreover, CB1 receptor agonists enhance NAc DA release when administered systemically or in the VTA (Oleson et al., 2012; Covey et al., 2018), but suppresses DA release evoked by local stimulation in the NAc (Mateo et al., 2017). Thus, how CIN-evoked DA release and its suppression by CB1 receptors on PFC terminals associates with or drives distinct behaviors remains elusive. Additional questions remain regarding CB1 receptor control of terminal DA release. CB1 receptors are expressed on axon terminals of glutamatergic inputs from the PFC, BLA, and vHPC (Grueter et al., 2010; Deroche et al., 2020), as well as GABAergic FSIs (Wright et al., 2017), but how these inputs, or their modulation by eCBs, controls terminal DA release is not known.

Emerging evidence also indicates that CB2 receptor signaling locally modulates NAc DA release. Unlike CB1Rs, CB2Rs do not alter Ca^2+^ or inwardly rectifying K^+^ channels (Felder et al., 1995). While CB2 receptors have historically been thought to reside primarily in the periphery, with high expression levels in the spleen and immune cells, more recent work has identified CB2 receptors throughout the brain, including the midbrain and striatum (Jordan and Xi, 2019). In contrast to CB1 receptors, CB2 receptors are expressed by DA neurons and their activation inhibits DA neuronal firing (Zhang et al., 2014). Moreover, both systemic and local administration of the CB2 agonist JWH133 into the NAc dose-dependently reduces extracellular DA levels measured with microdialysis (Xi et al., 2011; Zhang et al., 2017). CB2R activation also inhibits DA release in dorsal striatal slices through an mAChR M4-dependent mobilization of 2-AG from D1-MSNs onto CB2 receptors, which are presumably located on DA terminals (Foster et al., 2016). The ability to negatively regulate NAc DA transmission supports the CB2 receptor as a potential target for drug addiction therapy (Jordan and Xi, 2019).

### Non-canonical Neuromodulator Control of DA Release

In addition to “classic” neurotransmitters (e.g., glutamate, GABA, Ach), several neuropeptides control DA neurotransmission via alterations in vesicular DA release, transporter-mediated uptake, or degradation. Although MSNs are typically categorized by DA receptor expression (i.e., D1- versus D2-type), MSNs are also differentiated by which endogenous opioid they produce (Castro and Bruchas, 2019). D1-MSNs produce dynorphin (DYN) that functions as the endogenous ligand for the κ-opioid receptors (KORs) while D2-MSNs produce enkephalin (ENK) that binds both Δ-opioid receptors (DORs) and μ-opioid receptors (MORs). Opioid receptors are inhibitory GPCRs (i.e., G_*i/o*_–coupled) that suppress neuronal function and are expressed throughout the NAc. KORs are expressed on DA terminals (Svingos et al., 2001) and reduce DA transmission by suppressing vesicular release probability and DA synthesis, and increasing the rate of DAT-mediated DA uptake (Thompson et al., 2000; Britt and McGehee, 2008). In contrast, DOR activation generally increases DA release in the NAc through circuit interactions potentially involving inhibition of KOR signaling (Svingos et al., 1999). MORs are the majority of cell types in the NAc and their activation can increase or decrease NAc DA release through a variety of direct and indirect actions (Britt and McGehee, 2008; Gomez et al., 2019).

Low threshold spiking interneurons also control DA release via neuropeptide signaling through the release of SOM and expression of NPY receptors. Infusions of SOM into the NAc potently increases dialysate DA levels, possibly by potentiating NAc glutamate release (Pallis et al., 2001). Similar to SOM, NPY infusions into the NAc elevate extracellular DA levels (Sorensen et al., 2009), produce a place preference (Brown et al., 2000), and increase motivation to seek out and consume sucrose (van den Heuvel et al., 2015). While NPY and SOM influence NAc DA dynamics and behavior, the direct mechanism remains elusive.

Insulin provides another form of neuropeptide control of DA release. Insulin receptors (IRs) are receptor tyrosine kinases that are densely expressed on the majority of CINs (96%) in the NAc, with limited expression on DA axons (Stouffer et al., 2015). IR binding on CINs increases CIN firing and potentiates DA release via a nAChR binding on DA axons (Stouffer et al., 2015). Somewhat paradoxically, IR binding also increases DA uptake rate, which diminishes the potentiation in vesicular DA release (Stouffer et al., 2015). Notably, insulin regulation of NAc DA release depends on diet, such that IR signaling increases DA release during food restriction, but loses efficacy following a high fat diet (Stouffer et al., 2015).

## Conclusions, Caveats, and Future Directions

In this review, we described how varied cell types in the NAc are modulated by numerous afferent projections and local neuromodulators. Yet, how these complex neural circuit mechanisms ultimately influence behavior remains unclear. “Traditional” neural manipulation techniques (e.g., lesions or pharmacology) demonstrate a crucial role for the NAc in goal-directed action, but these approaches are generally unable to isolate specific cell types and circuits. More recent optogenetic developments that permit manipulations or measurements of genetically defined cell types represent an important advancement in differentiating the heterogenous signaling modalities in the NAc. However, these approaches generally still lack the requisite resolution for identifying how individual cells, their afferent inputs, and local neuromodulators interact in a spatiotemporally specific manner to control output targets and influence behavior. First, synchronized neuronal manipulations may identify that a target population *can* affect behavior, but this likely does not reflect how separate neurons with unique firing patterns and distinct synapse-specific connections (i.e., neuronal “ensembles” expanded upon in section “Characterization and Manipulation of Defined Neuronal Ensembles”) normally control behavior. This is clearly highlighted by work showing that optogenetic activation of D1- or D2-MSNs (Hikida et al., 2010, 2013; Lobo et al., 2010; Koo et al., 2014; Soares-Cunha et al., 2020) or FSIs (Chen et al., 2019) in the NAc can promote reward or aversion depending on the stimulation pattern (see Table 1). Moreover, any neuronal population is unlikely to be deterministic in isolation, and direct activation or inhibition of targeted cell types does not reflect normal modes of receptor-mediated synaptic transmission, which occurs across varied spatial and temporal scales on a variety of receptor systems. Finally, genetic profile is not deterministic. Spatially intermixed neurons may display a similar molecular profile, but possess distinct input-output connectivity and perform functionally heterogeneous roles (see section “Characterization and Manipulation of Defined Neuronal Ensembles”). Below, we will briefly discuss recent advancements that are building upon existing technology to better understand neural circuit control of behavior.

### Fluorescent Detection and Modulation of Neuronal Excitation

The degree to which neural manipulation techniques inform how the brain controls behavior relies on the fidelity by which these approaches mimic endogenous neural mechanisms. A number of genetically encoded fluorescent sensor techniques have been developed that can inform neural manipulation approaches by permitting cell type- and circuit-specific monitoring of numerous neural signaling mechanisms. We refer the reader to excellent reviews that offer a more in-depth discussion on this topic (Wang et al., 2018; Sabatini and Tian, 2020). These techniques rely on genetically modified fluorescent proteins that emit light following a conformational change in response to a specific cell signal, such as a change in membrane potential, ion flux, intracellular signal transduction, or receptor binding. Fluorescent detection of electrical signaling in the brain is accomplished using genetically encoded voltage indicators (GEVIs) (Knopfel and Song, 2019). GEVIs are able to provide information-rich readouts of continuous hyperpolarizing and subthreshold depolarizing signals with subcellular resolution. However, a number of technical hurdles related to adequate signal-to-noise ratios and stable expression in the plasma membrane of live brain tissue currently limits their widespread use in behaving animals. Alternatively, genetically encoded Ca^2+^ indicators (GECIs) such as GCaMPs are widely used *in vivo* to measure intracellular Ca^2+^ flux, which is often interpreted as a proxy of cell firing or neurotransmitter release (Pal and Tian, 2020). Similar intracellular signaling sensors have been developed for monitoring second messenger signals, such as kinases or GTPases (Rost et al., 2017). In recent years, a large number of receptor-based sensors that convert ligand binding into fluorescence emission have been developed to study neurochemical signaling (Sabatini and Tian, 2020). These tools dramatically expand the scope of neurochemical sensing technology, offering rapid detection of numerous ligands. Continued development and implementation of fluorescent sensors with improved signal-to-noise ratios, faster kinetics, and expanded spectral range will permit sensitive and simultaneous detection of various neural signaling mechanisms during behavior.

While improved neural monitoring technology can theoretically better inform cell manipulation approaches, it is important to consider the differences between what is being measured and manipulated. For example, measures of Ca^2+^ flux are not a direct readout of action potential generation, which is often what is targeted with common optogenetic manipulations (e.g., ChR2). Another major issue with interpreting direct manipulations of neuronal function (e.g., opto- or chemogenetic approaches) is that these methods bypass normal modes of synaptic neurotransmission. This is particularly important because most neurons–including all classes of NAc neurons–release multiple neuromodulators that act on various receptor subtypes, making it difficult to identify the neural effector (e.g., SOM/NPY/NO+ LTSIs). Recent developments allow spatially- and temporally-resolved control of cell type-specific receptor signaling mechanisms, permitting investigation into the interplay between intracellular signaling and synaptic function (Spangler and Bruchas, 2017). For example, optogenetically activated chimeric G-protein coupled receptors (Opto-XRs) express a modified, light-sensitive extracellular receptor binding domain but a conserved intracellular loop, allowing anatomically- and temporally-resolved control of endogenous signaling mechanisms using light (Airan et al., 2009; Siuda et al., 2015). The ability to manipulate a specific receptor in a defined cell type using the opto-XR approach will likely prove particularly useful for elucidating eCB control of neural circuit function.

Another technique involving modified receptor-based technology includes the inducible Tango (iTango) approach that links light- and ligand-dependent receptor activity with inducible signaling pathways to drive gene expression (Kim et al., 2017; Lee et al., 2017). With this approach, coincident light delivery and endogenous ligand binding lead to the release of an intracellular transcription factor that is designed to drive protein expression in cells that bind the ligand of interest. This can be used, for example, to express fluorescent proteins in cells that bind a specific ligand during a particular event, creating a snapshot of neural signaling during that period. Alternatively, the engineered transcription factor can drive expression of an excitable opsin or a caspase to then identify how neurons activated during the event of interest influence behavior. A number of additional optogenetic-based methods have been developed to directly target intracellular processes, even within specific organelles (Rost et al., 2017).

### Characterization and Manipulation of Defined Neuronal Ensembles

Cell type-specific genetic techniques are powerful tools for probing spatially intermixed cell populations. However, the use of widespread molecular markers to target an entire region or cell type within a region (e.g., glutamate- or GABA-expressing neurons), regardless of their activity state during the behavior of interest, likely masks the underlying neural ensembles–consisting of a small percentage of the target population–that allow animals to discern the complexity of their environment. The idea that distinct neuronal subpopulations control specific neural functions and behaviors stems from work on “engrams,” defined as enduring physical changes elicited by learning that underlie memory formation (DeNardo and Luo, 2017). This work typically uses immediately early genes (IEGs) such as *Fos* and *Arc* that are expressed within minutes following neuronal activation, but only in a small population within a particular brain region (∼2–12%) (Cruz et al., 2013). Activated IEG promoters can be targeted using pharmacology (Koya et al., 2009) or viral-genetic approaches (Zhou et al., 2019) to drive protein expression for labeling or manipulating neurons activated by a particular event (e.g., drug exposure, fear conditioning). In the NAc, this approach has demonstrated that a small population of accumbal neurons controls the hyperlocomotor or reinforcing effects of cocaine (Koya et al., 2009; Zhou et al., 2019). Viral strategies that use synthetic, activity-dependent promoters to drive IEG expression in genetically defined cell types allow improved isolation of active subpopulations (Bobadilla et al., 2020). Activity-dependent labeling of DA neurons may elucidate physiological factors that control neuronal and behavioral responses to certain events (e.g., drug exposure, stress) and promote pathological states.

### From Cells to Circuits to Treatments

While a cell type- and circuit-specific understanding of NAc function is a noble endeavor, how such an understanding would influence neuropsychiatric therapy is unclear. It is currently not possible to treat patients with manipulations that alter brain function with cellular resolution. However, understanding how precise neural signaling systems in specific brain regions control valence-based actions may improve treatment protocols. For example, deep brain stimulation (DBS) is a treatment that involves targeted delivery of electricity to a select brain region using an implanted electrode. The NAc is a target for DBS treatment in multiple conditions including obsessive compulsive disorder (OCD) (Koya et al., 2009), treatment-resistant bulimic anorexia nervosa (Fernandes Arroteia et al., 2020), and substance use disorders (Hassan et al., 2020). While the precise mechanism by which DBS relieves symptoms remains elusive, an improved understanding of how NAc circuitry controls of behavior may inform more targeted manipulation of specific neural elements or circuits at distinct time points in conjunction with pharmacotherapies. For example, eCB manipulations along with NAc DBS may improve outcomes by filtering a subpopulation of synaptic connections that are affected by stimulation. Moreover, cell- or ensemble-specific experiments may identify cellular markers that promote pathophysiology (e.g., psychiatric symptoms, neurodegeneration), potentially leading to more targeted therapeutics.

## Author Contributions

Both authors listed have made a substantial, direct and intellectual contribution to the work, and approved it for publication.

## Conflict of Interest

The authors declare that the research was conducted in the absence of any commercial or financial relationships that could be construed as a potential conflict of interest.

## Publisher’s Note

All claims expressed in this article are solely those of the authors and do not necessarily represent those of their affiliated organizations, or those of the publisher, the editors and the reviewers. Any product that may be evaluated in this article, or claim that may be made by its manufacturer, is not guaranteed or endorsed by the publisher.
